# Asymmetric Plasmonic Moth-Eye Nanoarrays with Side Opening for Broadband Incident-Angle-Insensitive Antireflection and Absorption

**DOI:** 10.3390/ma16175988

**Published:** 2023-08-31

**Authors:** Rong Xia, Yang Li, Song You, Chunhua Lu, Wenbin Xu, Yaru Ni

**Affiliations:** 1 State Key Laboratory of Materials-Oriented Chemical Engineering, College of Materials Science and Engineering, Nanjing Tech University, Nanjing 210009, China; 2 Jiangsu Collaborative Innovation Center for Advanced Inorganic Function Composites, Nanjing Tech University, Nanjing 210009, China; 3 Jiangsu National Synergetic Innovation Center for Advanced Materials (SICAM), Nanjing Tech University, Nanjing 210009, China; 4 Beijing Institute of Environmental Features Science and Technology on Optical Radiation Laboratory, Beijing 100854, China

**Keywords:** broadband antireflection, angle insensitive, surface plasmonic resonance, asymmetric nanostructure

## Abstract

Plasmonic absorbers with broadband angle-insensitive antireflection have attracted intense interests because of its wide applications in optical devices. Hybrid surfaces with multiple different sub-wavelength array units can provide broadened antireflection, while many of these antireflective surfaces only work for specific angles and require high complexity of nanofabrication. Here, a plasmonic asymmetric nanostructure composed of the moth-eye dielectric nanoarray partially modified with the top Ag nanoshell providing a side opening for broadband incident-angle-insensitive antireflection and absorption, is rationally designed by nanoimprinting lithography and oblique angle deposition. This study illustrates that the plasmonic asymmetric nanostructure not only excites strong plasmonic resonance, but also induces more light entry into the dielectric nanocavity and then enhances the internal scattering, leading to optimized light localization. Hence, the asymmetric nanostructure can effectively enhance light confinement at different incident angles and exhibit better antireflection and the corresponding absorption performance than that of symmetric nanostructure over the visible wavelengths, especially suppressing at least 16.4% lower reflectance in the range of 645–800 nm at normal incidence.Moreover, the reflectance variance of asymmetric nanostructure with the incident angle changing from 5° to 60° is much smaller than that of symmetric nanostructure, making our approach relevant for various applications in photocatalysis, photothermal conversion, and so on.

## 1. Introduction

Plasmonic absorbers, such as metallic nanostructures, have attracted wide attention due to their unique optical properties of localized field enhancement and enhanced light-mater interaction, which can boost the optical performance in various application, such as solar energy harvesting, photovoltaic devices, photo-catalysis and so on [1,2,3,4]. However, not all incident light contributes to the surface plasmonic resonance (SPR) due to undesired reflection loss at the interface between the incident medium and the metal surface, which severely limits the performance of these plasmonic absorbers. Great efforts have been made to design novel antireflective surfaces with the optimized tunability of an antireflective wavelength range and low reflectivity [5,6,7]. Multilayers with high and low refractive index film stacks and patterned nanostructures with sub-wavelength array units are wildly used to obtain broadband antireflection. The antireflective wavelength range of multilayers can be broadened by combining different layers [8,9], and patterned hybrid nanostructures by placing two or more nanostructures together can also provide broadband antireflection [10,11]. In practical applications, reducing the reflection and improving the absorption of light from wide angles of incidence in a broad wavelength range are crucial for enhancing the performance of optical, optoelectronic, and electro-optical devices [12,13,14,15,16]. However, many antireflective surfaces reported only operate well for a limited range of incident angles close to normal incidence. Essential problems are encountered for an oblique angle of incidence: there is a center resonant wavelength shift, and the antireflection gradually deteriorates for higher incident angles on account of the poor impedance match as the incident angle increases [17,18], making it unable to meet all the stringent requirements suitable for the aforementioned applications.

Recently, metal-insulator-metal (MIM) nanostructures, which consist of—top metallic nanoarrays and a ground metal layer separated by a thin dielectric spacer, have been explored to improve angle-independent antireflection [19,20]. The antireflective MIM nanostructures can be classified into two categories. The first kind utilizes the extraordinary transmission effect to reduce the amount of reflective losses. For instance, Zhao et al. designed a MIM grating opening an extraordinary transmission window for a seamless metallic layer and achieved above 70% transmission even under a large-angle incidence [21]. Cai et al. proposed a hybrid dielectric (Si and SiO_2_) 2D grating stacked on a continuous gold film to obtain high transmission (above 80%) over the 550 nm wavelength width [22]. The second kind takes advantage of the effect of SPR, leveraging strong light-trapping to enhance light absorption and improve angle insensitivity [23,24,25]. Zhu et al. reported plasmonic nanovoid arrays by nanosphere assembly, annealing, and multiple depositions providing reflection below 1.2%, even under a large incident angle of 60° [26]. Toma et al. demonstrated a gold nanocone array on flexible Teflon films to exhibit low reflectivity (below 1%) and strong absorption (around 90%) at a wide angle (0–70°) [27]. Dai et al. investigated a double-sided polarization-independent plasmonic absorber showing a high absorbance over a wide incident-angle range at the near-infrared region [28]. However, the single plasmonic MIM structures providing angle insensitivity can only work at a specific wavelength due to their narrow-band SPR [29]. Inspired by the cornea of moths, many sub-wavelength biomimetic quasi-moth-eye structures providing a gradient refractive index, such as nanopyramids, nanocones and nanonipples, are investigated as broadband antireflection structures [3,30,31]. Combining moth-eye nanostructures with the antireflective MIM nanostructures can integrate the gradient-refractive-index effect and SPR effect, which is promising to produce enhanced broadband and angle-insensitive antireflection and absorption performance.

**Figure 1:**
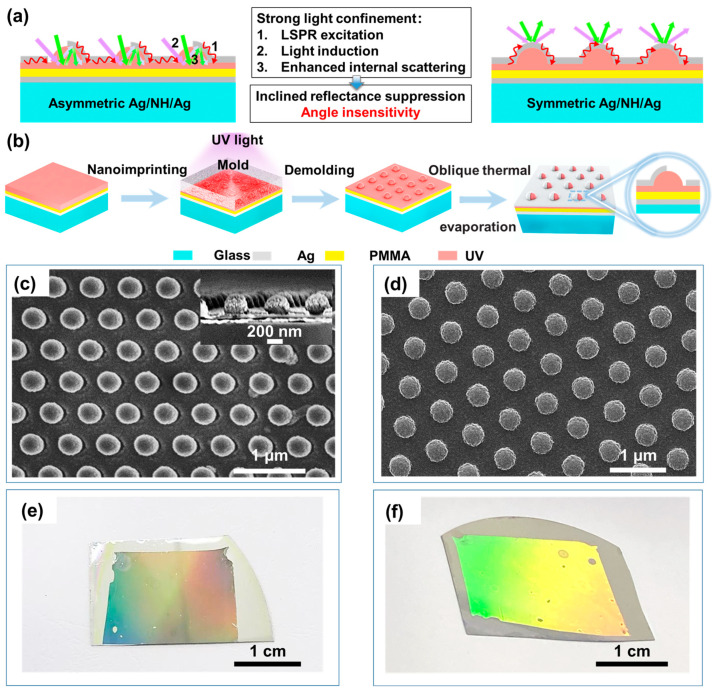
Schematic illustration of (**a**) asymmetric Ag/NH/Ag and symmetric Ag/NH/Ag. (**b**) The nanofabrication process of asymmetric Ag/NH/Ag. SEM images of (**c**) asymmetric Ag/NH/Ag and (**d**) symmetric Ag/NH/Ag. Photographs of (**e**) asymmetric Ag/NH/Ag and (**f**) symmetric Ag/NH/Ag under exposure to the fluorescent lamp.

In this work, to achieve incident-angle-insensitive broadband antireflection and absorption, we report on a plasmonic asymmetric nanostructure, which is composed of an asymmetric silver nanoshell and a planar silver film, sandwiching the photoresist nanohemispherical (NH) arrays (Figure 1a), fabricated by combining nanoimprinting lithography and oblique angle deposition. The moth-eye NH arrays with a gradient refractive index create a slowly varied transition from free space into nanostructures, reducing the reflection in the visual light range caused by the abrupt changes in the refractive index [32,33]. The asymmetric Ag nanoshell on the one hand provides the LSPR, which contributes to the direct absorption of incident light. On the other hand, the side opening of the asymmetric Ag nanoshell provides the prerequisite for additional light introduction from different incident angles and multiple points for internal scattering within NH nanocavities in order to significantly improve angle insensitivity. Compared to the symmetric Ag/NH/Ag, the incident light is better confined to the asymmetric Ag/NH/Ag, which is responsible for the desirable reflectance suppression over the incidence-angle range of 5°–60° over 300–800 nm, demonstrating outstanding broadband and angle-insensitive antireflection. We believe the improved antireflection and absorption properties provided by the proposed nanostructure will be helpful for many optical applications, like photothermal conversion, and will help increase optical system efficiencies.

## 2. Materials and Methods

### 2.1. Materials

Polymethyl methacrylate (PMMA) was obtained from Sigma-Aldrich (St. Louis, MO, USA). Chlorobenzene purchased from Macklin was the solvent for dissolving the PMMA. The UV-curable resist was used to prepare NH arrays.

### 2.2. Fabrication of Symmetric Ag/NH/Ag and Asymmetric Ag/NH/Ag

First, a 50-nm-thick silver reflecting layer was deposited onto a glass substrate by thermal evaporation. Next, the PMMA solution was spin coated on the silver film at 3000 rpm for 45 s and baked at 80 °C on a hot plate for 2 min. Then, the UV-curable resist was spin coated on the sample at 2000 rpm for 45 s, and a hexagonal nanohole mold was pressed into it. The mold contact was made on one edge and then gently rolled across the sample to completely remove any bubbles. The UV-curable resist was solidified by exposure to a UV source in a nitrogen atmosphere for 8 min, ensuring a fully cured imprint. The Ag/NHs were fabricated after the mold was gently removed. Finally, 40 nm Ag film was deposited onto the aforementioned nanostructures by normal thermal evaporation to fabricate symmetric Ag/NH/Ag, while 40 nm Ag film was deposited onto Ag/NH by oblique thermal evaporation to fabricate asymmetric Ag/NH/Ag.

### 2.3. Characterization

The designed structures were observed with a scanning electron microscope (SEM) at acceleration voltages of 3 and 15 keV. The roughness and surface topography of the structures were characterized by atomic force microscopy (AFM) on an MM8-SYS scanning probe microscope (Bruker AXR, Billerica, MA, USA). The diffuse reflectance spectra of designed structures in the wavelength range of 300–800 nm were measured by an ultraviolet–visible spectrometer (Cary 5000, Agilent, Santa Clara, CA, USA) equipped with an integrating sphere. The angular dependence of the optical properties was measured using a variable-angle reflectance accessory.

### 2.4. Numerical Simulation

The simulations were conducted utilizing the commercial software of Lumerical finite-difference time-domain (FDTD) solutions to investigate the optical properties of designed structures. The optical constants of silver were taken from Werner, and the refractive indices of PMMA and UV were extracted by ellipsometry. The sizes of designed structures wereestimated from the SEM images. In modeling, the periodicity of the FDTD boundary along x- and y-directions was 600 nm and √3 × 600 nm, respectively. Moreover, perfectly matched layer (PML) boundary conditions were set along the z- direction. For the sake of guaranteeing the convergence and stability of the simulations, the high mesh accuracy size of 2 nm was adopted. For normal incidence, the periodic plane wave of 300–800 nm in wavelength was implemented.

## 3. Results and Discussion

### 3.1. The Fabrication of Asymmetric Ag/NH/Ag

**Figure 2:**
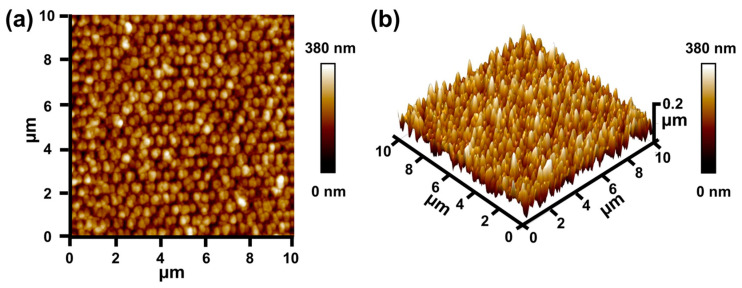
(**a**) Two- and (**b**) three-dimensional AFM images of asymmetric Ag/NH/Ag.

Different kinds of technologies have been developed to produce the moth-eye nanostructures, including optical lithography [34,35], colloidal lithography [10,11,36], and nanoimprint lithography [37,38]. However, the first two methods require additional reactive ion etching (RIE) processes to form the moth-eye nanostructures, which is inconvenient and costly for large-area processes. Nanoimprint lithography can transfer the pattern from the reusable mold using direct contacting and resin curing, which does not require additional expensive optical equipment [39,40]. By contrast, nanoimprint lithography is comparatively cost effective and easy to fabricate with high repeatability. Hence, in this work, as seen in Figure 1b, asymmetric Ag/NH/Ag is obtained by the following nanofabrication process, based on nanoimprint lithography and oblique thermal evaporation (Figure 1b). (a) A 50-nm-thick silver reflecting layer is firstly deposited onto a glass substrate by thermal evaporation, and then a 50-nm-thick PMMA layer and 70-nm-thick UV-curable resist layerare successively formed on the silver film by spincoating. (b) The UV-curable resist layer is then patterned via nanoimprinting by attaching a PDMS soft mold on it, and cured by UV exposure. After the demolding process, the hexagonal array of NH on Ag with a feature size of 250 nm and period of 600 nm can be achieved. (c) Finally, 40 nm Ag film is deposited onto the aforementioned nanostructures when an unequal angle of 45° has been employed in oblique thermal evaporation, leading to the asymmetric silver nanoshell (Figure 1c). Compared with the symmetric Ag/NH/Ag (Figure 1d), which is obtained by nanoimprinting and normal thermal evaporation, asymmetric Ag/NH/Ag is more elliptical from the top view, with a major diameter of 290 nm and minor diameter of 260 nm. The AFM 2D and 3D images included in Figure 2, which further exhibit the morphology of asymmetric Ag/NH/Ag, show a hexagonal-packed nanohemisphere feature array with some interspaces, consistent with the SEM observation. All of these images confirm that the asymmetric Ag/NH/Ag has been successfully fabricated. In Figure 1e,f, the reflected light can be clearly seen from the surface of symmetric Ag/NH/Ag, whereas reflected light on the surface of asymmetric Ag/NH/Ag is dramatically reduced.

### 3.2. The Broadband Antireflection of Asymmetric Ag/NH/Ag

**Figure 3:**
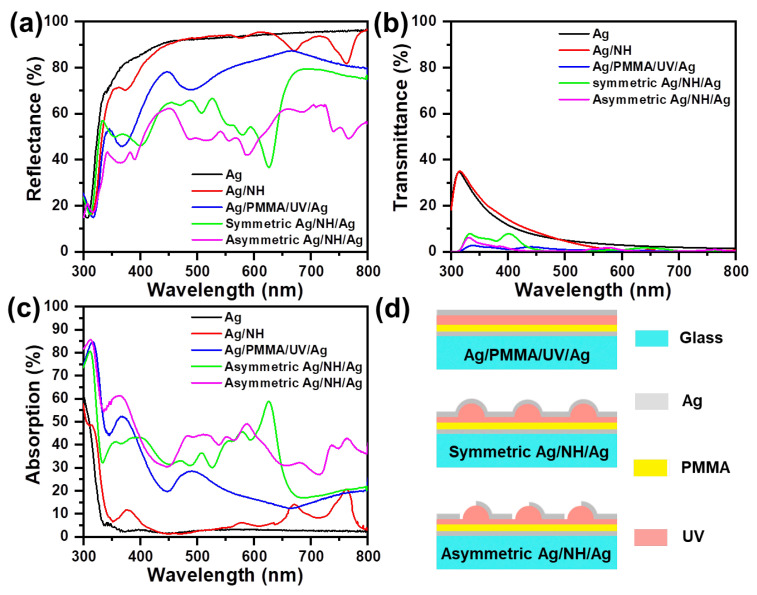
(**a**) Diffuse reflectance (measured by an ultraviolet–visible spectrometer equipped with an integrating sphere), (**b**) transmittance spectra and (**c**) absorption spectra of designed nanostructures at normal incidence: Ag film, Ag/PMMA/UV/Ag film, Ag/NH, symmetric Ag/NH/Ag, and asymmetric Ag/NH/Ag. (**d**) Schematic illustration of Ag/PMMA/UV/Ag, symmetric Ag/NH/Ag, and asymmetric Ag/NH/Ag.

To investigate the antireflective behavior of asymmetric Ag/NH/Ag, we analyze the reflectance spectra comparison of these nanostructures: flat Ag film, Ag/NH, flat Ag/PMMA/UV/Ag film, symmetric Ag/NH/Ag and asymmetric Ag/NH/Ag, as schematically shown in Figure 3a. The reflectance spectrum of the Ag/NH exhibits two slight dips around 671 and 763 nm, with the reflectance of 87.3% and 81.8%, respectively. After adding a 40 nm Ag layer to the Ag/NH by normal thermal evaporation, the reflectance of the wavelength from 336 to 780 nm is all obviously reduced by an average of 26.3%. Therein, the most apparent deep valley (626 nm) of symmetric Ag/NH/Ag reflectance spectrum exhibits 45.1% decreased reflectance compared with the reflectance valley of Ag/NH at 763 nm. The antireflective performance of symmetric Ag/NH/Ag is probably due to the excitation of the SPR and thus brings about strong resonance.

**Figure 4:**
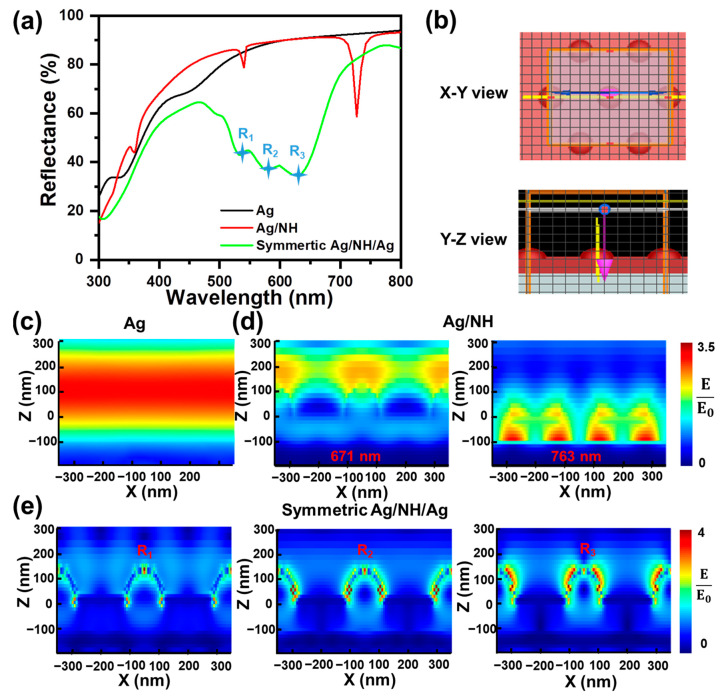
(**a**) Numerically calculated reflectance spectra of designed nanostructures: Ag, Ag/NH and symmetric Ag/NH/Ag. (**b**) Schematic illustration of simulation area. Electric field distributions of (**c**) Ag film at 600 nm, (**d**) Ag/NH, and (**e**) symmetric Ag/NH/Ag at resonant wavelengths.

In order to further verify the abovementioned SPR effect on the antireflective performance of symmetric Ag/NH/Ag, we simulate the spectra, as well as the cross-sectional electric field intensity profiles (Figure 4), at the main resonant wavelengths of Ag film, Ag/NH, and symmetric Ag/NH/Ag using the FDTD method. In both cases, good agreement is observed between FDTD predictions and experimental measurements. Slight deviation originating from experimental variations is acceptable. According to the simulated electric field profile of Ag/NH, at the reflectance valley corresponding to the wavelength of 671 nm, the majority of the electric field is mainly distributed around the external edges of the NH top, which is probably due to the excitation of the periodic lattice resonant (PLR). At the wavelength of 763 nm, the electric field is mainly concentrated inside the NH arrays and is the strongest at the dielectric section near the Ag/NH interface, indicating the coupled excitation of the SPR and PLR mode. The electric field distributions of Ag/NH/Ag at the wavelength corresponding to the main deep valleys (R_1_: 535 nm, R_2_: 578 nm, R_3_: 626 nm), which show a similar profile, generate the strongest localization at the external Ag surface of the NH, manifesting the excitation of the coupled PLR and SPR modes. The higher concentration of the electric field of symmetric Ag/NH/Ag compared to that of Ag/NH probably elicits strong resonance absorption and substantially improves antireflection.

Further breaking of the geometric symmetry of the top Ag nanoshell by introducing a breach on one sidewall opens a channel for light entering into the NH nanocavity, which gives rise to more reflectance valleys in the range of 300–800 nm. The average reflectance of asymmetric Ag/NH/Ag in the 320–610 nm and 645–800 nm ranges obviously decreases compared with symmetric Ag/NH/Ag; especially the reflectance of the wavelength from 645 to 800 nm is at least reduced by 16.4%. The significant reflection suppression can be attributed to the enhanced light absorption by its light-trapping structure. On the one hand, more light is induced into the NH nanocavity through the open channel of asymmetric Ag nanoshell. On the other hand, when light enters into the NH nanocavity, more multiple points for internal scattering are generated; thus, the interaction between light and the nanostructure is enhanced and substantially heightens SPR. Considering that the Ag/NH/Ag and asymmetric Ag/NH/Ag potentially affect both the multilayer interface and the SPR factor, we compare the flat Ag/PMMA/UV/Ag film with the abovementioned nanostructures. The reflectance of Ag/PMMA/UV/Ag film is lower than that of the Ag/NH, but obviously higher than that of symmetric Ag/NH/Ag and asymmetric Ag/NH/Ag. Moreover, we also measure the transmittance spectra of the designed nanostructures to analyze the absorption variation in Figure 3b. Since the transmittance of symmetric Ag/NH/Ag and asymmetric Ag/NH/Ag remains below 8%, and the transmittance difference remains small in the wavelength range of 300–800 nm, which further indicates the refection reduction of asymmetric Ag/NH/Ag is due to the stronger SPR and light scattering effect. According to the reflectance and transmittance spectra, we calculate the absorption of the designed nanostructures. It is obvious that the absorption of the asymmetric Ag/NH/Ag is enhanced compared to the symmetric Ag/NH/Ag, especially in the wavelength ranges of 460–610 nm and 645–800 nm. Based on the above analysis, it can be deduced that the incident light is better confined inside asymmetric Ag/NH/Ag, and the antireflective amplitude is obviously heightened by utilizing an asymmetric Ag nanoshell with an open channel due to the synergistic interaction of the light introduction effect, strong excitation of the SPR, and enhanced internal scattering.

### 3.3. The Incident Angle Insensitivity of Asymmetric Ag/NH/Ag

**Figure 5:**
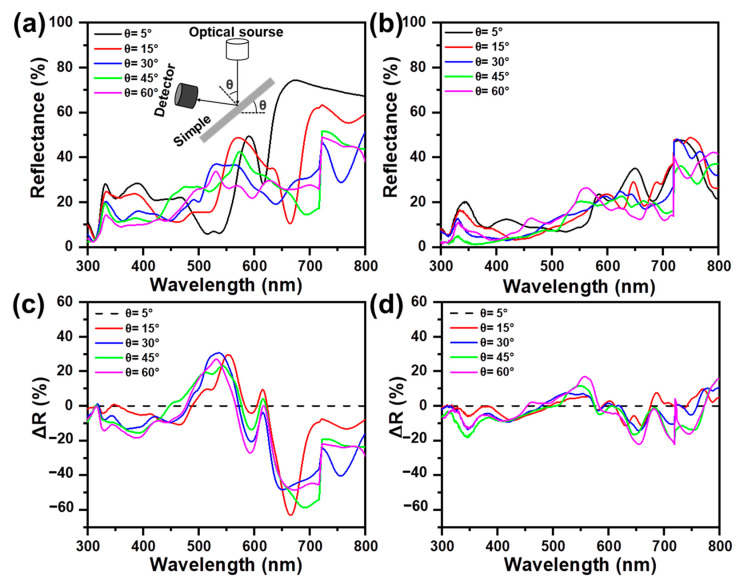
Specular reflectance spectra of (**a**) symmetric Ag/NH/Ag and (**b**) asymmetric Ag/NH/Ag measured with different incident angles (θ= 5°, 15°, 30°, 45°, 60°).The differences (ΔR) between the reflectance measured at a 5° incident angle and that at other incident angles (θ= 15°, 30°, 45°, 60°) of (**c**) symmetric Ag/NH/Ag and (**d**) asymmetric Ag/NH/Ag.

To observe the impact of incident angles on the antireflective performance of asymmetric Ag/NH/Ag, the specular reflectance comparison between symmetric Ag/NH/Ag and asymmetric Ag/NH/Ag with various incident angles (θ) varying from 5° to 60° are investigated in Figure 5a,b. As for symmetric Ag/NH/Ag, the main reflectance valleys obviously shift with the incidence angles increasing, and thus, complex oscillations in the reflectance spectrum can be observed, showing significant angle-dependence on the incidence angles. In contrast, the reflectance spectrum of asymmetric Ag/NH/Ag shows a relatively smaller shift and exhibits a lower reflectance (<40%) over the wavelength range of 300–720 nm with the incidence-angle changing from 0° to 60°. For a more intuitive presentation, the difference (∆R) between the reflectance at the other incident angles and the incident angle of 5° is exhibited in Figure 5c,d. The symmetric Ag/NH/Ag shows significant dependence on the incidence angles, with ∆R ranging from −63.1% to 30.5%, while the reflectance variance of asymmetric Ag/NH/Ag with varied incident angles is much smaller, with ∆R ranging from −22.7% to 17.8%. Based on the above experimental results, it can be deduced that the incident light at different angles is better confined inside asymmetric Ag/NH/Ag by utilizing its Ag nanoshell, which is because the incident light at oblique angles enters into the semi-closed nanocavity more easily and enhances internal light scattering simultaneously. Hence, the reflectance of asymmetric Ag/NH/Ag at different incident angles can be suppressed effectively, and angle insensitivity is realized.

### 3.4. The Optical Behavior Observed at Opposite Incident Angles of Asymmetric Ag/NH/Ag

**Figure 6:**
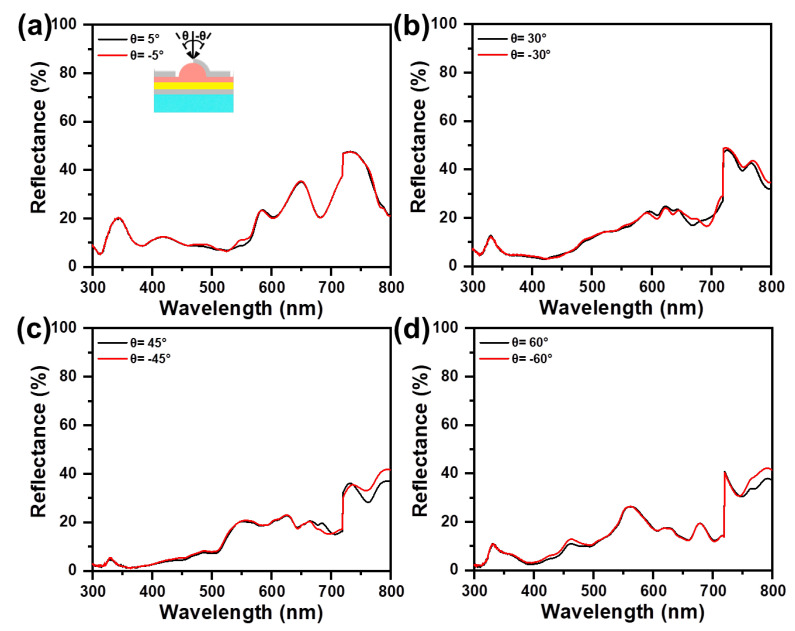
Specular reflectance spectra of asymmetric Ag/NH/Ag, measured with different incident angles of (**a**) θ = ±5°, (**b**) θ = ±30°, (**c**) θ = ± 45°, and (**d**) θ = ±60°.

**Figure 7:**
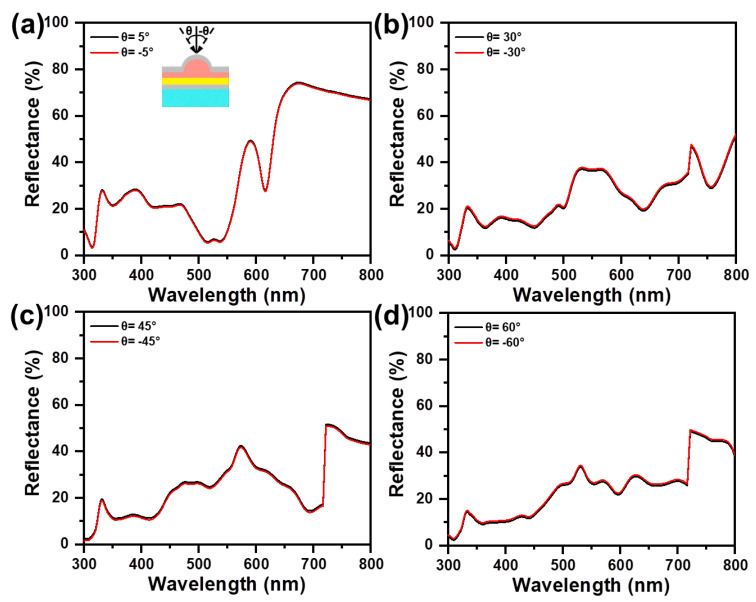
Specular reflectance spectra of symmetric Ag/NH/Ag, measured with different incident angles of (**a**) θ= ±5°, (**b**) θ= ±30°, (**c**) θ= ± 45°, and (**d**) θ= ±60°.

The evaporation of 40 nm Ag from an inclined angle leads to deposition at the side and the top of the underlying Ag/NH, which gives rise to a directional optical difference. Figure 6 shows the reflectance difference of asymmetric Ag/NH/Ag at the same incident angle (θ = 15°, 30°, 45°, 60°), but with opposite directions. Here, negative angles are those that are in the same direction as the metal evaporation. The spectral tendency measured from opposite incident angles performs a similar feature, whereas for negative angles the reflectance remains relatively higher. For the incident angle of ±5°, the difference between the reflectance with opposite incident directions is slight. With the incident angle increasing, the reflectance difference increases from 2.4% (θ = ±5°) to 5.6% (θ = ±60°), showing an amplified asymmetric optical performance between opposite incident angles. As to symmetric Ag/NH/Ag, the reflectance observed from the opposite incident angle remains the same at different oblique incidences (Figure 7). Although the primary results indicate that this asymmetric optical behavior can be amplified with structural asymmetry and oblique incidence, the deviation of the opposite reflectance, even at large incident angles, is relatively slight, manifesting that asymmetric Ag/NH/Ag exhibits the angle-insensitive property.

## 4. Conclusions

In this study, an asymmetric nanostructure based on a partially enclosed plasmonic nanocavity is prepared by nanoimprinting lithography and oblique angle deposition. The introduction of the asymmetric silver nanoshell can effectively suppress light reflection, especially at oblique incidences, due to enhanced absorption resulting from structural scattering and light confinement in the NH nanocavity. As a result, asymmetric Ag/NH/Ag provides lower reflectance than symmetric Ag/NH/Ag in the ranges of 320–610 nm and 645–800 nm; especially the reflectance of the wavelength from 645 to 800 nm is at least reduced by 16.4%. Meanwhile, the reflectance variance of the asymmetric Ag/NH/Ag with varied incident angles is much smaller than that of symmetric Ag/NH/Ag, with the ∆R range decreasing from 93.6% to 40.5%, demonstrating that asymmetric Ag/NH/Ag possesses weaker angular dependence of the optical response than symmetric Ag/NH/Ag. In addition, the asymmetric Ag/NH/Ag exhibits a slight difference with the opposite observing angle. This design realizes insensitive antireflection with varied incident angles in the range of 300–800 nm, which could have significant impacts and potential applications in photocatalysis, photothermal conversion, and so on.

## Data Availability

The data presented in this study are available on request from the authors.
